# Serum total antioxidant capacity reflects severity of illness in patients with severe sepsis

**DOI:** 10.1186/cc4826

**Published:** 2006-02-20

**Authors:** Chia-Chang Chuang, Shu-Chu Shiesh, Chih-Hsien Chi, Yi-Fang Tu, Lien-I Hor, Chi-Chang Shieh, Ming-Feng Chen

**Affiliations:** 1 Department of Emergency Medicine, College of Medicine, National Cheng Kung University, Tainan, Taiwan; 2 Institute of Clinical Medicine, National Cheng Kung University, Tainan, Taiwan; 3 Department of Medical Technology, College of Medicine, National Cheng Kung University, Tainan, Taiwan; 4 Department of Microbiology and Immunology, College of Medicine, National Cheng Kung University, Tainan, Taiwan; 5 Department of Internal Medicine, Show Chwan Memorial Hospital, Changhua, Taiwan; 6 Graduate Institute of Integration Chinese and Western Medicine, China Medical University, Taichung, Taiwan

## Abstract

**Introduction:**

We conducted the present study to evaluate the changes in serum total antioxidant capacity (TAC) in patients with severe sepsis and to investigate the association between serum TAC and clinical severity.

**Method:**

This was a prospective observational study involving a sample of patients who met established criteria for severe sepsis and were admitted to the emergency department of a university teaching hospital. Serum TAC was determined using the total radical-trapping antioxidant parameter method. The levels of TAC, uric acid, albumin, and bilirubin in sera were obtained in the emergency department and evaluated to determine whether there were any correlations between the major antioxidant biomarkers and clinical severity of sepsis. The Acute Physiology and Chronic Health Evaluation (APACHE) II score was used for clinical evaluation of the severity of sepsis.

**Results:**

A total of 73 patients with sepsis, with a mean (± standard deviation) APACHE II score of 23.2 ± 8.2 and a mortality rate of 26.0%, were included. Seventy-six healthy individuals served as control individuals. Among the patients, serum TAC levels correlated significantly with APACHE II scores. Patients who died also had higher TAC than did those who survived. Serum uric acid levels correlated significantly with serum TAC and APACHE II scores in patients with severe sepsis.

**Conclusion:**

Elevated serum TAC level may reflect clinical severity of sepsis. In addition, serum uric acid levels appear to contribute importantly to the higher TAC levels observed in patients with severe sepsis.

## Introduction

Severe sepsis is a challenging problem in the emergency department or intensive care unit (ICU) [1], and can lead to septic shock or multiple organ failure. The complex mechanisms underlying severe sepsis remain unclear. In sepsis, the overwhelming inflammatory response to the invading pathogen is the major pathophysiologic challenge, rather than the pathogen itself. In a systemic inflammatory response, both endothelial cells and neutrophils are activated to release oxygen-derived free radicals [2]. It seems that these oxyradicals play a role in causing or propagating the systemic inflammatory response syndrome (SIRS) in life-threatening conditions, and that the imbalance in redox state reflects both oxidative stress and tissue damage [3,4].

Measurement of serum total antioxidant capacity (TAC) level was reported to provide an integrated index, as opposed to one based on simple summation of measurable antioxidants [5]. It possibly could be used to assess the real change in antioxidant status in patients with severe sepsis and might lead to universally useful treatment [6]. Several preclinical and clinical studies of sepsis focused on single-point inhibition (for example, anti-tumor necrosis factor antibodies) or augmentation of specific key processes, and failed to demonstrate therapeutic efficacy [7]. Most believe that higher levels of oxyradicals and lower antioxidant levels in patients with SIRS or septic shock lead to multiple organ failure [8-10]. However, serum TAC increases in critically surgical patients with septic shock [11]. Moreover, endogenous peroxyl-radical scavenging ability in the plasma of SIRS patients was found to be elevated in nonsurvivors [12]. The actual change in TAC in severe sepsis remains controversial.

Serum uric acid (UA), like other antioxidants such as albumin, bilirubin, or vitamins A, C and E, is a powerful free-radical scavenger and increases in response to acute oxidative stress [11,13]. UA formation may even provide a significant antioxidant defense mechanism against nitration by peroxynitrite in rat heart during hypoxia [14]. It is therefore postulated that serum UA level is an important marker in oxidative stress. Recently, serum UA was identified as a strong predictor of mortality in patients with moderate-to-severe chronic heart failure [15]. This finding raises an interesting question about the actual pathophysiologic role of serum UA in critically ill patients.

We conducted the present study to investigate whether serum TAC levels are elevated or suppressed in emergency department patients with severe sepsis. We also wished to determine the correlation between serum TAC level and severity of illness, and the relationship between serum TAC and UA levels in emergency department patients with severe sepsis.

## Materials and methods

The study was conducted between April 2001 and March 2003 at an academic tertiary care center, which receives between 43,000 and 54,000 emergency department visits each year. The study was approved by our hospital's Institutional Research Review Board.

### Patients selection

Patients aged 18 years old or older meeting criteria for severe sepsis (including septic shock) were recruited into the study, after consent had been obtained from the relatives of the patients. The inclusion criteria used for severe sepsis were those defined by the American College of Chest Physicians/Society of Critical Care Medicine Consensus Conference [16]. 'Severe sepsis' is defined as sepsis associated with organ dysfunction, hypoperfusion abnormality, or sepsis-induced hypotension. 'Septic shock' is a subset of severe sepsis and is defined as sepsis-induced hypotension that persists despite adequate fluid resuscitation combined with hypoperfusion/organ dysfunction [16]. Patients with noninfectious diseases such as acute coronary syndrome, acute stroke, acute pancreatitis, drug intoxication, and severe renal dysfunction (serum creatinine >3.0 mg/dl) were excluded. All patients were first evaluated in the emergency department and were then observed throughout the duration of the admission to identify possible sources of infection. We also included 76 healthy control individuals, selected from those undergoing an annual health examination, who were matched to the patients with respect to age and sex.

### Laboratory determinations

Peripheral venous blood samples available in the emergency department were put into sterile collection tubes without anticoagulant and centrifuged at 3,500 rpm for 15 minutes. The supernatants were aliquoted into Eppendorf tubes and stored at -70°C until analysis.

### Measurements of serum total antioxidant capacity and other serum biomarkers

Serum TAC, UA, albumin, and bilirubin were measured as indicators of antioxidative status. Serum levels of TAC and UA were determined within six hours after a patient had arrived in the emergency department. Serum TAC was assessed using the total radical-trapping antioxidant parameter (TRAP) method [17] and on a luminometer (AutoLumat LB 953; EG & G Berthold, Bad Wildbad, Germany) to determine the TAC level, as previously described [18]. Briefly, a chemiluminescent reaction was generated in a collection tube by carefully mixing 800 μl distilled water, 100 μl signal reagent (luminol and p-iodophenol in buffer solution), and 50 μl 1:200 diluted horseradish peroxidase solution (Sigma, St. Louis, MO, USA). Then, 10 μl of the sample was added to inhibit the luminescence. The duration of quenching was measured and compared with that of water-soluble ascorbic acid. The precision of the assay (coefficient of variation) was 2.3% for within-day variation and 5.1% for day-to-day variation. Serum concentrations of UA, albumin, and bilirubin were determined using commercial kits and an automated biochemical analyzer (Hitachi 747; Roche Diagnostics, Mannheim, Germany).

### Evaluation of clinical severity and primary outcome

The Acute Physiology and Chronic Health Evaluation (APACHE) II score [19] is used to evaluate the severity of disease. APACHE II score was the first system to use a quantitative evaluation of disease severity in the ICU [20], and the score was calculated within 24 hours of emergency department admission. The primary outcome was whether the serum TAC correlated with APACHE II score in patents with severe sepsis. The secondary outcome was 28-day in-hospital mortality. In order to evaluate the relative contribution of serum TAC to patient outcome, patients were divided into 'survivors' and 'nonsurvivors'. Survivors were those patients who were still alive 28 days after admission, including an ICU stay; nonsurvivors were patients who died within 28 days of emergency department admission.

### Statistical analysis

All values were expressed as the mean ± standard deviation. Descriptive statistics for TAC, UA, albumin, bilirubin, APACHE II score, length of stay, and age were recorded and analyzed using SPSS for Windows 11.5 (SPSS, Chicago, IL, USA). Prevalence and associated 95% confidence intervals were calculated using conventional methodology [21]. The comparisons among serum TAC levels and among groups with different APACHE II scores were made using one-way analysis of variance and post-hoc comparisons ('least significant difference'). Spearman rank nonparametric correlation was used to estimate the correlation between TAC levels and each of UA, albumin, bilirubin, and APACHE II score. Comparisons between survivors and nonsurvivors were conducted using the Mann-Whitney U exact test. *P *< 0.05 was considered statistically significant. Multiple linear regressions were used to assess the associations between serum TAC levels and APACHE II scores after controlling for covariates such as age and serum creatinine levels.

## Results

The study enrolled 149 participants aged over 18 years: 73 patients who met the criteria for severe sepsis (including septic shock), as defined by the American College of Chest Physicians/Society of Critical Care Medicine Consensus Conference; and 76 healthy control individuals. These patients (41 men and 32 women; mean age 65.9 ± 16.4 years) presented with a mean APACHE II score of 23.2 ± 8.2; the mortality rate was 26.0% (19 out of 73; Table 1). Of the patients, 39 (53.4%) had pneumonia, nine (12.3%) had soft tissue infections (including necrotizing fasciitis and Fournier gangrene, among others), seven (9.6%) had urinary tract infections, six (8.2%) had biliary tract infections, three (4.1%) had central nervous system infections (including brain abscess, meningitis, among others), and nine (12.3%) had unknown foci of infection. Nonsurvivors had higher APACHE II scores and a higher ratio of septic shock in the emergency department than did survivors. The leading infectious micro-organism were Gram-negative bacteria (35.6%) and the positive culture rate was 75.3% (55 out of 73). Detailed demographic data, clinical diagnoses, and microbiological data for patients and healthy control individuals are summarized in Table 1.

### Correlation between serum total antioxidant capacity level and clinical severity

**Figure 1:**
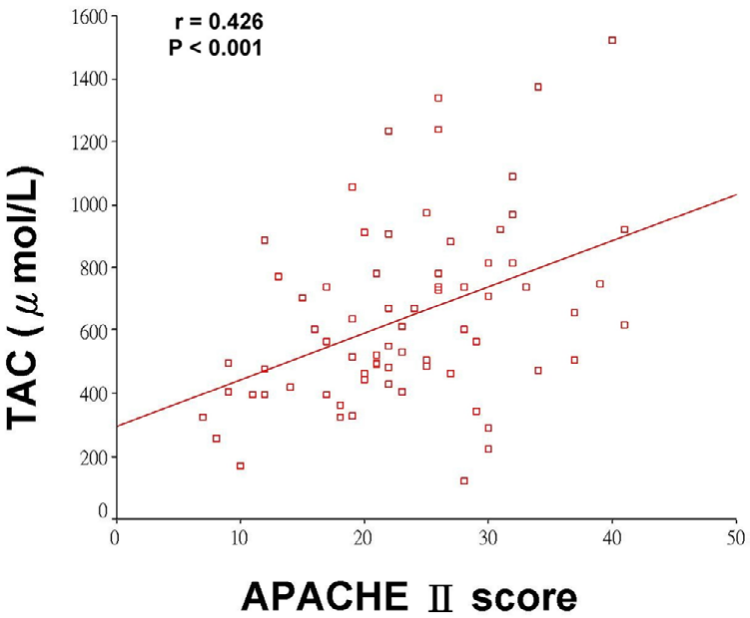
Correlation between serum TAC and APACHE II score in patients with severe sepsis. A total of 73 patients were included in the study. APACHE, Acute Physiology and Chronic Health Evaluation; TAC, total antioxidant capacity.

Serum TAC levels in patients with severe sepsis correlated positively with APACHE II scores (*r *= 0.426, 95% confidence interval [CI] 0.2–0.6; *P <*0.001; Figure 1). After controlling for age and serum creatinine level, TAC still exhibited a positive correlation with APACHE II score (*P *= 0.027).

### Comparison of serum total antioxidant capacity levels in healthy control individuals and patients

**Figure 2:**
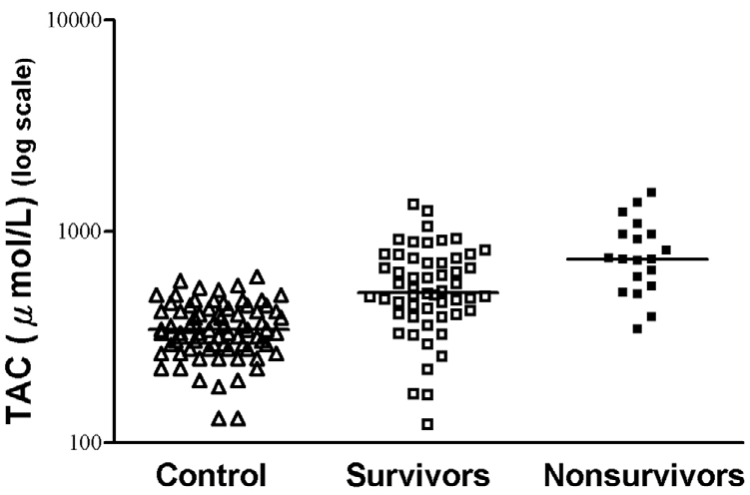
Serum TAC in control and patients (stratified by outcome). The trial included a total of 76 healthy control individuals; of the 73 patients, 54 survived ('survivors') and 19 died ('nonsurvivors'). The horizontal bars represent the mean level for each group. TAC, total antioxidant capacity.

Serum TAC levels were significantly higher in patients with severe sepsis than in healthy control individuals (637.0 ± 290.9 μmol/L versus 355.2 ± 102.7 μmol/L, 95% CI 211.7–351.9; *P <*0.001). Furthermore, serum TAC levels were higher in nonsurvivors than in survivors (812.0 ± 322.4 μmol/L versus 575.4 ± 254.6 μmol/L, 95% CI 91.2–382.0; *P = *0.002; Figure 2).

### Correlation between serum total antioxidant capacity and uric acid, bilirubin, and albumin levels in patients

**Figure 3:**
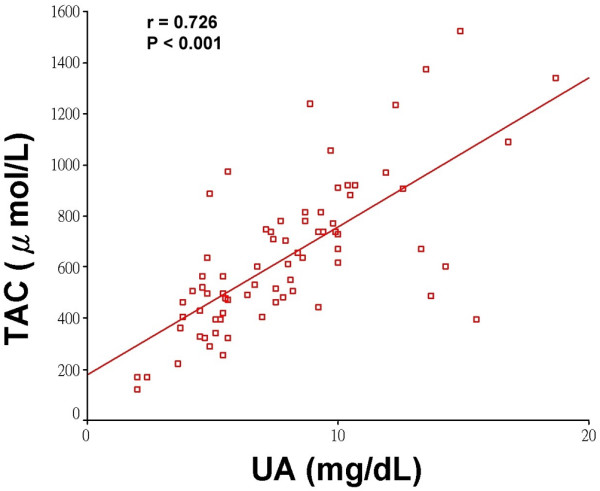
Correlation between serum TAC and UA in patients with severe sepsis. A total of 73 patients were included in the study. TAC, total antioxidant capacity; UA, uric acid.

Serum TAC levels in patients with severe sepsis were significantly and positively correlated with serum UA levels (*r *= 0.726, 95% CI 0.595–0.819; *P <*0.001; Figure 3). No correlation was found between serum TAC and albumin level. Serum TAC levels exhibited a weak but significant correlation with bilirubin levels (*r *= 0.311, 95% CI 0.1–0.5; *P = *0.02).

### Correlation between serum uric acid and clinical severity in patients

**Figure 4:**
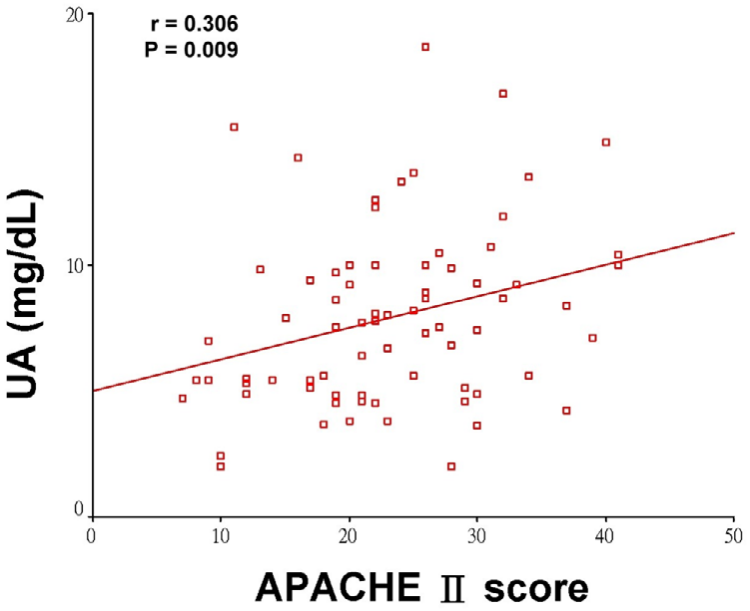
Correlation between serum UA and APACHE II score in patients with severe sepsis. A total of 73 patients were included in the study. APACHE, Acute Physiology and Chronic Health Evaluation; UA, uric acid.

Serum UA exhibited a weak but significant correlation with APACHE II score (*r *= 0.306, 95% CI 0.082–0.501; *P *= 0.009; Figure 4). However, there was no significant correlation of serum albumin or bilirubin level with APACHE II score.

## Discussion

In this study we showed that serum TAC levels reflected clinical severity of sepsis. Serum TAC levels became elevated as APACHE II scores rose. In addition, serum TAC levels were higher in nonsurvivors than in survivors. Why serum TAC increased in patients with severe sepsis is unknown. It is hypothesized that anti-inflammatory processes are activated to counterbalance excessive levels of proinflammatory cytokines [22] or oxidative stress [23]. It is also possible that altered antioxidant defense (i.e. a significant change in serum TAC level) eventually leads to immune dysfunction and a poor outcome [11,12,24].

Higher sustained serum TAC levels, as noted in the nonsurvivors, might be a host response to severely propagating oxidative stress or a compensating mechanism for depleted antioxidative components [13]. It is also possible that the increase in serum TAC simply worsened severe sepsis, which suggests that antioxidant therapy should not routinely be used in the treatment of severe sepsis until its mechanism of action is understood.

TAC levels in patients with severe sepsis remain controversial. Although Ghiselli and coworkers [5] suggested that plasma rather than serum should be used to measure TAC levels, in the study by Whitehead colleagues [17] and in ours [18] serum was used. In one of our other experiments there appeared to be no significant difference between the levels detected in plasma and serum (data not shown). Pascual and coworkers [11] reported that plasma TAC levels were lower in patients with sepsis but higher in patients with septic shock than in control individuals. MacKinnon and colleagues [13], in a study conducted in 50 critically ill patients, reported that total antioxidant status and UA levels were higher in nonsurvivors than in survivors, and speculated that the higher total antioxidant status level might have reflected the higher UA levels caused by renal dysfunction. In accordance with our findings, Tsai and coworkers [12] reported that plasma TAC levels were significantly higher in nonsurvivors than in survivors. However, most other studies [2,8,25] found that the plasma antioxidant potentials of nonsurvivors were significantly lower than those of survivors among patients with severe sepsis.

The discrepancy in the findings from these studies may mainly be due to differences in measurement methods. Instead of using a spectrophotometric method [2], we and two other groups [11,12] used the TRAP method to measure the total antioxidant potential. The spectrophotometric method, proposed by Miller and coworkers [26], measures the reaction of plasma antioxidants and a mixture of different radical species including 2,2'-azinobis(3-ethylbenzothiazoline 6-sulfonic acid; ABTS) radical cation, ferryl myoglobin, hydrogen peroxide, and other radicals. In the spectrophotometric methods, albumin and UA account for 43% and 33%, respectively, of TAC. The marked decrease in serum albumin in patients with severe sepsis may lead to lower levels of TAC detected using the spectrophotometric method. On the other hand, the TRAP method measures the reaction of total antioxidants and peroxyl radicals generated by 2,2'-azobis(2-amidinopropane hydrochloride; ABAP) [27]. The contribution of albumin to the TRAP reaction is very low (<10%), whereas that of UA is higher (47–57%, but even up to 76.4%). Therefore, change in serum UA may influence serum TAC levels determined using the TRAP method.

Our findings also indicate a significant increase in serum UA and a positive correlation between serum UA and TAC in patients with severe sepsis and septic shock. In addition, serum UA levels correlated significantly with APACHE II scores. Similarly, a study of neonatal sepsis [28] found that serum UA concentrations were increased by 51% in babies with septic shock as compared with control babies. Jabs and coworkers [29] also found that plasma UA levels increased in relation to higher APACHE II scores in critically ill patients. All of these findings suggest that serum UA is an important contributor to serum TAC, which in turn may reflect the clinical severity of severe sepsis. The mechanism of increased UA in patients with severe sepsis and septic shock is unknown. Both increased production and decreased excretion of UA may result in elevated serum level. Severe sepsis and septic shock may induce ischemia or hypoxia in multiple organs, which further increases the change in xanthine/hypoxanthine to UA through activation of xanthine oxidase in microvascular endothelium [30,31]. On the other hand, renal dysfunction induced by septic shock may reduce the secretion of UA from the kidneys, which may increase serum UA and further elevate serum TAC [5,13]. In the present study, we excluded patients with previous renal dysfunction and patients undergoing hemodialysis. Therefore, our finding of increased serum UA or TAC in patients with severe sepsis or septic shock could not have been a consequence of renal failure. Whether hyperuricemia is a risk factor for severe sepsis is unknown. Our findings simply demonstrate that serum UA levels were correlated with APACHE II scores, and indicate that hyperuricemia might be associated with poorer clinical outcomes in sepsis. In this study elevation of serum UA could not represent TAC completely. More studies are needed to investigate the mechanism underlying the relation between serum UA and sepsis.

## Conclusion

Serum TAC levels were elevated in severe sepsis, especially in nonsurvivors, and were positively correlated with clinical severity. Serum UA, which contributes largely to TAC level, plays an important role in elevating serum TAC in patients with severe sepsis. Further studies are needed to confirm our observations and elucidate the underlying mechanisms.

## Key messages

• 	Overall, serum total antioxidant capacity levels were higher in patients with severe sepsis, especially in nonsurvivors.

• 	Serum total antioxidant capacity could reflect clinical severity.

• 	Furthermore, serum uric acid levels contributed largely to high total antioxidant capacity levels.

## Abbreviations

APACHE = Acute Physiology and Chronic Health Evaluation; CI = confidence interval; ICU = intensive care unit; SIRS = systemic inflammatory response syndrome; TAC = total antioxidant capacity; TRAP = total radical-trapping antioxidant parameter; UA = uric acid.

## Competing interests

The authors declare that they have no competing interests.

## Authors' contributions

CCC conceived the study and drafted the manuscript. CCC, LIH, and MFC all contributed to the study design. CCC obtained research funding. SCS and MFC supervised the conduct of the trial and data collection. CHC and YFT recruited patients, oversaw bacteriology, and managed the data. SCS and CCS analyzed the data, including quality control. Professor Shin-Tair Wang provided statistical advice on study design. All authors contributed substantially to its revision and approved the final manuscript. MFC takes responsibility for the paper as a whole.

## Figures and Tables

**Table 1:** Characteristics of patients with severe sepsis and of healthy control individuals

	Patients with severe sepsis		
		
Characteristic	Survivors (*n *= 54)	Nonsurvivors (*n *= 19)	Healthy controls (*n *= 76)
Age (years)	65.1 ± 17.0	68.4 ± 14.6	65 ± 7.9
Gender (male/female)	29/25	12/7	43/33
APACHE II score	21.0 ± 7.3	29.4 ± 7.8*	-
Septic shock (emergency department visit)	27 (50.0%)	15 (78.9%)*	-
Length of stay in ICU (days)	29.2 ± 20.2	9.7 ± 11.0*	
Diagnoses of severe sepsis (*n *[%])			
Pneumonia	29 (53.7)	10 (52.6)	
Soft tissue infections	8 (14.8)	1 (5.3)	
Urinary tract infections	6 (11.1)	1 (5.3)	
Biliary tract infections	5 (9.3)	1 (5.3)	
Central nervous system infections	1 (1.8)	2 (10.5)	
Unknown foci	5 (9.3)	4 (21.0)	
Microbiological data (*n*)			
Gram-negative bacteria	21	5	
Gram-positive bacteria	12	3	
Polybacterial	8	2	
Fungi	3	0	
Positive cultures^a^	45	10	
Positive bacteremias^b^	20	5	

Data are shown as mean ± standard deviation, unless otherwise stated. *P < 0.05, versus survivors. ^a^Rates of positive culture; samples include sputum, urine, and other tissues. ^b^Rates of positive blood culture.
